# An examination of single day vs. multi-day heart rate variability and its relationship to heart rate recovery following maximal aerobic exercise in females

**DOI:** 10.1038/s41598-020-71747-8

**Published:** 2020-09-08

**Authors:** Emily Bechke, Brian Kliszczewicz, Cherilyn McLester, Mark Tillman, Michael Esco, Roxanna Lopez

**Affiliations:** 1 University of North Carolina at Greensboro, 1400 Spring Garden St., Greensboro, NC 27412 USA; 2 Kennesaw State University, 520 Parliament Garden Way NW, Kennesaw, GA 30144 USA; 3 The University of Alabama, 101 8th St., Tuscaloosa, AL 35401 USA

**Keywords:** Electrophysiology, Stress and resilience, Autonomic nervous system

## Abstract

The purpose of this study was to examine the relationship of a single day measure of heart rate variability (HRV), and the averaged baseline measures of HRV to heart rate recovery (HRR) following maximal exercise. Thirty females (22.9 ± 3.2 years, 64.8 ± 8.4 kg) completed four visits (V1–V4), where a 10-min HRV was recorded. Upon completing the V4 recording, a treadmill graded exercise test (GXT) was performed, followed by a 5-min active cool down. HRV was assessed through time domain measures [natural log of root mean square of successive R–R differences (lnRMSSD) and standard deviation of normal to normal intervals (lnSDNN)] and natural log frequency domain measures [low frequency (lnLF) and high frequency (lnHF)]. Variables collected over V1–V4 were measured as; day of (DO) GXT, 3 day (AV3), and 4 day average (AV4). HRR was calculated as the maximal HR achieved minus the HR at: 30-s (HRR30), 1-min (HRR1), 2-min (HRR2), 3-min (HRR3), 4-min (HRR4) or 5-min (HRR5) of recovery. Pearson’s Product correlations revealed significant correlations (*P* = < 0.05) between all HRV_DO_ measures to each HRR measure and are presented in ranges: lnSDNN (r = 0.442–0.522), lnRMSSD (r = 0.458–0.514), lnLF (r = 0.368–0.469), lnHF (r = 0.422–0.493). For HRV_AV3,_ lnRMSSD_AV3_ and HRR1 were positively correlated (r = 0.390, *P* = 0.033). Last, HRV_AV4_ showed positive relationships (*P* = < 0.05) between lnRMSSD_AV4_ and HRR30 (r = 0.365, *P* = 0.048); and for HRR1 and lnSDNN_AV4_ (r = 0.400, *P* = 0.029), lnRMSSD_AV4_ (r = 0.442, *P* = 0.014), and lnHF_AV4 (_r = 0.368, *P* = 0.045); and lnRMSSD_AV4_ and HRR3 (r = 0.381, *P* = 0.038). Within the current study HRV_DO_ displayed the strongest correlations to HRR therefore, averaged resting HRV measures do not strengthen the prediction of cardiovascular recovery following a GXT in this population.

## Introduction

The indirect evaluation of the autonomic nervous system (ANS) is performed through the assessment of cardiovascular metrics such as heart rate variability (HRV) and heart rate recovery (HRR)^1–3^. Heart rate variability quantifies the timing between consecutive beat-to-beat intervals and provides an indirect measure of systemic ANS activity^2,4^. In applied settings, resting HRV is used as an indicator of physiological readiness and recovery by estimating or predicting the ANS’s ability to respond to demands placed on the system^2,5,6^. More specifically, the higher the variability the greater the parasympathetic nervous system (PNS) influence (i.e., greater recovery status), whereas depressed HRV values are indicative of increased sympathetic nervous system (SNS) activity (i.e., lower recovery status)^7,8^. In addition to HRV, HRR is used to examine autonomic function and/or vagal tone reactivation following submaximal and maximal exercise^1,4,9^ in order to provide insight into ANS health. Heart rate recovery is commonly calculated as the change in peak heart rate (HR) achieved during submaximal or maximal exercise from HR at specific time points during recovery (e.g. 30 s, 1-min, etc.)^4^. Segmental measurements of HRR can be broken down into two separate phases, a fast phase and a slow phase. The fast phase (30 s to 2-min) represents PNS reactivation whereas the slow phase (2- to 5-min) is suggestive of the combined activity of PNS reactivation and SNS withdrawal^4,9,10^.

Together, HRV and HRR provide wider insight to cardiac autonomic regulation which can then translate to clinical or applied applications. The shared system of regulation between HRV and HRR suggest that the two measures may be interrelated. If such a relationship exists, resting HRV indices may be used as a predictor of HRR (ANS rebound) prior to a stressful stimulation such as exercise and potentially reduce the risk of clinical testing or reduce periods of overreaching in athletic populations. Thus, the relationship between resting HRV to post exercise HRR has shown to be a promising area of evaluation; however equivocal results have been found^11–16^. The apparent disconnect within the literature between resting HRV and HRR may be explained through procedural differences, study population, analysis techniques, or exercise and recovery protocols^4^. Further investigations should be performed to aid in the deduction of the various findings. Although there are numerous possibilities that may contribute to the conflicting findings within the literature, the use of a single resting measure of HRV showed to be a commonality between them. It is important to note that HRV, by nature, is a highly sensitive measure to factors such as quality of sleep, stress, and the environment which may lead to a change in day-to-day fluctuations within HRV indices. With these known influencers of HRV, a single day HRV measure may not provide a comprehensive representation of ANS status^5,17^. In order to account for potential day-to-day fluctuations, the use of multiple baseline values of HRV have been proposed and utilized in other areas of HRV research to provide a more precise estimate of autonomic function within an individual or population^5,18^.

The majority of research evaluating the use of averaged HRV indices over a series of days is primarily focused on guided training within athletic populations^5,18–20^. Recent studies by Plews et al., found that averaged resting HRV values over several days to be a better predictor of recovery status and tool to guide training versus a single day measure^19–22^. As the research advances with the use of averaged HRV indices and guided training, it becomes evident that these approaches may carry over to other physiological applications such as evaluating acute stress and recovery (e.g., exercise). To the authors’ knowledge, only one study has examined a potential relationship between averaged HRV baseline measures and HRR^16^. Tonello et al. examined 21 apparently healthy overweight women (34.5 ± 6.4 years of age) where HRV was collected for four consecutive days in a seated and standing position. Heart rate recovery was also collected over four consecutive days and analyzed at 1, 2, 3, and 5 min following submaximal exercise. Despite the use of averaged HRV measurements, no significant correlations were observed between HRR and HRV^16^. Although Tonello et al.^16^ had a novel approach to examine day-to-day HRV and HRR, it is currently unknown the relationship between averaged HRV measures on HRR following a single exercise bout within young healthy females. Therefore, the purpose of this study was to examine the relationships between a single day, a 3-day average, and a 4-day average baseline of HRV and HR measurements to HRR following a maximal aerobic exercise test in young healthy females.

## Methods

### Participants

Prior to the collection of any data, the Kennesaw State University Institutional Review Board approved all testing procedures and protocols, and all experiments were performed in accordance with relevant guidelines and regulations. A convenience sample of thirty-four apparently healthy, young apparently healthy females (22 ± 3 years of age, 64.9 ± 8.4 kg, 161.3 ± 6.6 cm) were recruited from the local metropolitan area. Each individual was made aware of the procedures and potential risks associated with the study and signed an informed consent prior to participation. Inclusion criteria required participants to be classified as “low risk” for exercise participation as defined by the guidelines of the American College of Sport Medicine^23^. Participants also filled out a health history questionnaire and any individual who reported having orthopedic conditions, or cardiovascular, pulmonary, or metabolic disease was excluded from the study. Prior to all sessions, participants were asked to wear clothing suited for exercise that was light and comfortable, fast for a minimum of 4 h (except for water consumption), avoid exercise and alcohol for 24-h, and avoid caffeine consumption for 12-h.

### Experimental design

**Figure 1:**
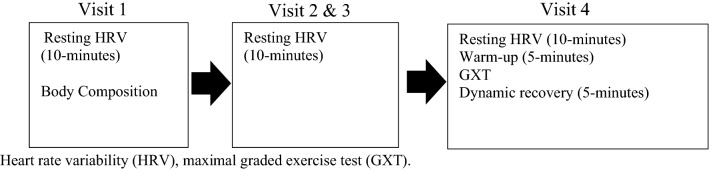
Study design. *HRV* heart rate variability, *GXT* maximal graded exercise test.

Participants reported to the institution’s exercise physiology laboratory on four separate occasions where the first three visits occurred within a 7 day period of each other and the fourth visit occurred within 7 days of the third visit. All visits were conducted between the hours of 4 a.m.–9 a.m. and were performed as close to wake time as possible. Visits one (V1), two (V2), and three (V3) were designed to collect resting cardiac autonomic measures, whereas visit four (V4) was used to collect cardiovascular measures prior to and following a maximal GXT. Upon arrival to V1–V3, baseline resting cardiac autonomic measures were collected using an advanced heart rate monitor for 10-min in the supine position. At the completion of the resting period, the visit ended. These procedures held true for V1–V3 with the exception of V1, where anthropometric measures were collected. Upon arrival to V4, resting cardiovascular measures were collected using the same procedures as V1–V3. At the completion of the resting period, participants performed a maximal GXT on a motorized treadmill. Immediately after the GXT participants began a standardized 5-min active cool down (see Fig. 1).

### Cardiac autonomic measures

Heart rate and HRV were recorded with the Polar Team^2^ system (Polar, Lake Success, NY). Each heart rate monitor was fitted to the participant and positioned directly over the sternum and was in direct contact with skin. All resting cardiac autonomic measures were recorded in a quiet, dimly lit room at an average temperature of 21.6 ± 0.9 °C and 53.6 ± 10.7% humidity. Each resting period took place in the supine position for 10-min and participants were instructed to breathe at their normal respiratory rate and to remain as still as possible until further instructed.

Heart rate variability and resting heart rate (RHR) were analyzed in 5-min segments, where the first 5-min of the recordings were discarded in order to allow for an adjustment period. Thus, only the last 5-min of the resting period were analyzed for HRV and RHR. Measurements of HRV were represented and analyzed as a single day measurement collected on the day of the maximal exercise test on V4 (DO), the average of V1–V3 (AV3), and the average of V1–V4 (AV4), respectively. Time and frequency domain measures were analyzed using Kubios software version 3.0 (Kubios V 3.0, Joensuu Finland) and set to an interpolation rate of 4 Hz. In order to detect the presence of artifact or noise, Kubios software used the piecewise cubic spline interpolation method to filter the data with a “very low-low artifact correction” and a sensitivity set to identify any R–R abnormalities ± 0.35 s compared to the local average^24^. Segments were visually inspected by a trained member of the research team for the presence of irregular R–R intervals (i.e. ectopic beats) and if the presence of three or more irregular R–R intervals were included in a segment, that segment was deleted in order to avoid misinterpretation of analysis.

Time domain measures of HRV chosen for the current study included the root mean square of successive R–R differences (RMSSD) as an indication of PNS activity^25^ and the standard deviation of normal to normal intervals (SDNN)^2^. Time domain analysis occurred through the process of converting R–R intervals into a tachogram where the y-axis represents the time (ms) between consecutive intervals and the x-axis represents the number of beats over time^26^. In addition to the time domain measures, the frequency domain measures chosen for the current study included low frequency (LF) and high frequency (HF). Frequency domain measures were extrapolated through the Fast Fourier Transformation (FFT) algorithm with the window width set at 300 s and a 50% overlap. The algorithm identified LF as signals between 0.04 and 0.14 Hz and signals from 0.15 to 0.4 Hz as HF. Low frequency is established as a universal measure that indicates both PNS and SNS activity, whereas HF represents PNS activity^26^.

### Graded exercise test and active cool down

At the completion of the resting period during V4, participants were allotted 5-min to warm-up on a motorized Woodway treadmill (Woodway USA, Waukesha, WI) at a self-selected pace. Following the warm-up, participants were fitted to a Parvomedics Metabolic Cart (Parvomedics TrueOne 2400, Sandy, UT) in order to assess peak aerobic capacity (VO_2peak_). The GXT was performed at a self-selected pace where the grade of the treadmill increased 1% each minute of the test until the participant reached volitional fatigue. Blood lactate was assessed immediately upon completion of the GXT via finger stick and portable lactate analyzer (Lactate Plus, Nova Biomedical, Waltham, MA). Simultaneously, the treadmill was set at a walking speed of 1.5 mph and 2.5% grade^1^ and participants walked for 5 min in order to cool down. Heart rate recovery measures were collected during the cool down.

### Heart rate recovery

Heart rate recovery was assessed through the Polar Team^2^ system software and calculated as the maximal heart rate achieved during the test minus the recovering HR at 30 s (HRR30), 1 min (HRR1), 2 min (HRR2), 3 min (HRR3), 4 min (HRR4), and 5 min (HRR5) of recovery. These measures incorporated both phases of HRR, which include a fast phase (30 s to 2-min) that represents PNS reactivation and the slow phase (2–5 min) which represents a combination of PNS and SNS activity^9,10^.

### Statistical analysis

All data were entered and analyzed in SPSS 25 software (Chicago, Illinois, USA). Measurements of RMSSD (ms), SDNN (ms), LF (ms^2^), HF (ms^2^), and RHR (beats/min) were analyzed as a single day measurement (DO), the average of V1–V3 (AV3), and the average of V1–V4 (AV4). Prior to statistical analysis, all HRV measures were log transformed (ln) due to a violation in normality according to a Shapiro–Wilk Normality Test. Differences between DO, AV3, and AV4 within each HRV and RHR measure, respectfully, was examined through an analysis of variance (ANOVA) with a least square difference (LSD) post hoc applied in order to observe pairwise comparisons. Pearson product correlations were used to assess relationships between RHR measures (RHR_DO,_ RHR_AV3,_ RHR_AV4_), HRV measures as represented in the time domain (lnRMSSD_DO_, lnRMSSD_AV3_, lnRMSSD_AV4_, lnSDNN_DO_, lnSDNN_AV3_, lnSDNN_AV4_) and frequency domain measure (lnLF_DO_, lnLF_AV3_, lnLF_AV4_, lnHF_DO_, lnHF_AV3_, lnHF_AV4_) to the respected HRR measures; HRR30, HRR1, HRR2 HRR3, HRR4 and HRR5. Significance was set at *P *≤ 0.05. Results are presented as means and standard deviation (M ± SD).

## Results

A total of thirty apparently healthy females (22.9 ± 3.2 years, 161.3 ± 6.5 cm, 64.8 ± 8.4 kg, maximal aerobic capacity = 37.6 ± 6.6 ml/kg/min) completed the study. Four participants withdrew due to scheduling conflicts. Means and standard deviations of lnRMSSD, lnSDNN, lnLF, lnHF, RHR, HRR, as individual days and averaged visits are presented in Table 1. Pairwise comparisons revealed no significant differences between DO, AV3, AV4 measures of HRV (*P* > 0.05), however there were significant differences between DO, AV3, AV4 measures of RHR (*P* < 0.05). Pearson correlation values and significance are displayed in Table 2. Resting heart rate measured as DO, AV3, and AV4 were significantly negatively correlated to each time point of HRR (Table 2). Additionally, significant positive correlations were observed between all HRV_DO_ measures to HRR30, HRR1, HRR2, HRR3, HRR4, HRR5; lnSDNN (r = 0.442–0.522), lnRMSSD (r = 0.458–0.514), lnLF (r = 0.368–0.469), lnHF (r = 0.422–0.493), respectively (Table 2). A single significant positive correlation was seen in HRV_AV3_ between lnRMSSD_AV3_ and HRR1 (r = 0.390, *P* = 0.033,). Last, HRV_AV4_ showed significant positive relationships between lnRMSSD_AV4_ and HRR30; lnSDNN_AV4_, lnRMSSD_AV4_, lnHF_AV4_ and HRR1; and lnRMSSD_AV4_ and HRR3 (Table 2).

**Table 1:** Resting raw and log transformed measures of HRV.

Measure	V1	V2	V3	DO	AV3	AV4	CV% AV3	CV% AV4
SDNN (ms)	64.6 ± 32.8	57.6 ± 24.9	55.7 ± 22.6	55.5 ± 24.7	59.13 ± 23.51	58.2 ± 23.5	20.9 ± 11.5	21.6 ± 9.9
lnSDNN (ms)	4.05 ± 0.50	3.96 ± 0.45	3.93 ± 0.44	3.93 ± 0.44	3.98 ± 0.42	3.97 ± 0.40	5.38 ± 3.13	5.56 ± 2.68
RMSSD (ms)	75.8 ± 45.7	71.2 ± 39.7	67.0 ± 37.3	65.6 ± 38.3	70.71 ± 38.28	69.7 ± 37.2	21.8 ± 15.4	24.7 ± 12.5
lnRMSSD (ms)	4.16 ± 0.60	4.12 ± 0.55	4.01 ± 0.56	4.02 ± 0.61	4.11 ± 0.53	4.08 ± 0.53	5.49 ± 4.03	6.43 ± 3.61
LF (ms^2^)	2002.4 ± 3,048.4	1,281.0 ± 1,481.8	1,055.1 ± 1,144.1	1,296.0 ± 1,205.6	1,436.7 ± 1686.5	1,401.4 ± 1527.8	53.7 ± 23.8	54.9 ± 21.2
lnLF(ms^2^)	6.92 ± 1.16	6.71 ± 0.92	6.50 ± 0.97	6.78 ± 0.92	6.71 ± 0.85	6.72 ± 0.83	9.36 ± 5.14	9.16 ± 3.98
HF (ms^2^)	2,685.5 ± 1968.2	2,395.2 ± 2,369.3	1948.6 ± 1813.8	1882.6 ± 2,130.1	2,268.6 ± 2,102.4	2,215.5 ± 2060.2	46.01 ± 29.6	51.1 ± 24.7
lnHF(ms^2^)	7.32 ± 1.14	7.34 ± 1.02	7.15 ± 1.00	7.00 ± 1.14	7.26 ± 0.94	7.20 ± 0.94	1.40 ± 7.64	2.35 ± 7.61

*V1* Visit 1, *V2* visit 2, *V3* visit 3, *DO* measures day of maximal exercise test, *AV3* average of V1–V3, *AV4* average of V1–V4, *CV% AV3* coefficient of variance averaged V1–V3, *CV% AV4* coefficient of variance averaged V1–V4, *lnRMSSD* log transformed root mean square of successive R–R intervals, *lnSDNN* the standard deviation of successive normal to normal intervals, *lnLF* low frequency, *lnHF* high frequency.

**Table 2:** Pearson product correlation values of resting HRV measures represented as a single day and averaged measure to HRR time points.

	HRR30	HRR1	HRR2	HRR3	HRR4	HRR5
**lnSDNN**						
DO	0.452*	0.522^†^	0.442*	0.516^†^	0.489^†^	0.471^†^
AV3	0.290	0.336	0.181	0.239	0.211	0.166
AV4	0.345	0.400*	0.259	0.323	0.295	0.255
**lnRMSSD**						
DO	0.462*	0.514^†^	0.448*	0.512^†^	0.479^†^	0.458*
AV3	0.308	0.390*	0.272	0.310	0.268	0.246
AV4	0.365*	0.442*	0.333	0.381*	0.341	0.317
**lnLF**						
DO	0.368*	0.420*	0.383*	0.469^†^	0.465^†^	0.399*
AV3	0.246	0.238	0.057	0.151	0.155	0.084
AV4	0.290	0.300	0.146	0.246	0.251	0.172
**lnHF**						
DO	0.422*	0.481^†^	0.433*	0.493^†^	0.459*	0.431*
AV3	0.204	0.297	0.201	0.244	0.193	0.171
AV4	0.281	0.368*	0.283	0.332	0.284	0.259
**RHR**						
DO	− 0.486^†^	− 0.454*	− 0.455*	− 0.517^†^	− 0.547^†^	− 0.468^†^
AV3	− 0.442*	− 0.449*	− 0.417*	− 0.470^†^	− 0.492^†^	− 0.410*
AV4	− 0.469^†^	− 0.465^†^	− 0.443*	− 0.499^†^	− 0.523^†^	− 0.441*

*DO* Single day HRV, *AV3* average HRV of the first three visits, *AV4* average HRV measures of all four visits, *HRR30* heart rate recovery at 30 s, *HRR1* 1 min, *HRR2* 2 min, *HRR3* 3 min, *HRR4* 4 min, *HRR5* 5 min, *RHR* resting heart rate, *lnRMSSD* log transformed root mean square of successive R–R intervals, *lnSDNN* the standard deviation of successive normal to normal intervals, *lnLF* low frequency, *lnHF* high frequency. *P < 0.05, ^†^P < 0.01.

## Discussion

The purpose of this study was to examine the relationships between various baseline measures of HRV (DO, AV3, AV4) and HRR (30, 1, 2, 3, 4, 5) in young adult females following a maximal GXT. The authors hypothesized that HRV_AV4_ would result in a more robust significant positive relationship to measures of HRR than those measured as HRV_DO_ or HRV_AV3_. The primary findings of the study do not support this hypothesis, indicating HRV_DO_ possessed the strongest positive relationships with all HRR measures. Measures examined as HRV_AV3_ had the least amount of significant correlations with a single point of significance between lnRMSSD_AV3_ and HRR1. Compared to HRV_AV3_, more robust and varied relationships were observed between HRV_AV4_ and HRR measures, but remained weaker than those of HRV_DO_ (Table 2).

Despite common physiological factors between resting HRV and HRR, the complexity behind their relationship and influence upon each other are not fully understood^3,4,9,12–15,27,28^. Esco et al. examined this relationship and found no correlations in time (SDNN) or frequency domain measures (HFnu, LFnu:HFnu) of resting HRV to HRR (1,2) following a similar GXT and recovery protocol in 66 healthy males^12^. These findings align with Bosquet et al. who observed a lack of significant correlations between resting HRV measures and HRR following a GXT on a treadmill in well-trained athletes^29^. Javorka et al. also examined the relationship between standing HRV indices and HRR1 in 17 healthy males following an 8-min step test and saw no correlations between the two different cardiac autonomic measures^13^. The current investigation does not fully support these observations, as significant correlations between resting HRV (DO, AV3, AV4) and HRR were reported. This may in part be due to the differences in population observed; where in the current study recreationally active females participated compared to either males or athletic populations in the aforementioned studies. The significant correlations between HRV_DO_ and HRR in the current study agree with those of Nunan et al., who found moderate and positive relationships between parasympathetic measures of lnRMSSD to HRR (2,3), lnHF to HRR (1,2,3), and moderate negative correlations between lnLF:HF to HRR (1,2,3) following a GXT in both male and female subjects^15^. Additionally, a recent study by Molina et al.^14^, observed moderate negative correlations between resting PNS measures and moderate positive correlations in measures of combined PNS and SNS indices to HRR (3,5)^14^. The systems involved in regulating ANS function at rest and in response to exercise are complex and highly interactive resulting in a variety of measures used to interpret the various components of this system. Therefore, interpretations should take into consideration external and intrinsic factors (e.g., sex, body comp, training status, anxiety…) when comparing these markers.

The current study demonstrated a positive relationship between resting HRV and HRR which is equivocal to current literature^11–13,16,27^. Though it was out of the scope of this study to examine mechanisms related to the differences in HRV and HRR measures, other factors such as resting cardiovascular values, methodological approaches, and homogeneity of population may explain the differences found within the literature^4,12,14,16^. The driving theory behind the hypothesis of the current investigation was the possible limitation of using single day resting HRV measures as a baseline. Several investigations have revealed significant day-to-day fluctuations in HRV, suggesting that a single day measurement may not provide a precise representation of resting autonomic indices^5,19–21,30^. Research by Plews et al.^20^ found that rolling averages^22^ may provide more insightful information to a resting state with a 7-day rolling average as the accepted standard. Despite these recent findings, the use of single day HRV measurements prior to an exercise intervention or acute study is common practice^31–33^. To the authors’ knowledge, only one other study examined the relationship between averaged HRV measures and HRR^16^. Tonello et al.^16^ examined the relationship of a 4-day average resting HRV to HRR following a maximal exercise test in 21 apparently healthy overweight women (34.5 ± 6.4 years of age). In addition to a 4-day assessment of HRV, HRR was also assessed over 4 days following a submaximal cycle ergometer test. Interestingly, no correlations were observed between resting HRV_AV4_ and HRR^16^. The primary outcome variables of the study conducted by Tonello et al.^16^ varied from the current study and therefore the differences to the current investigation may be due to study design (i.e. submaximal cycle test vs treadmill GXT)^4^ and population.

The findings of the current study differ from Tonello et al.^16^ as significant relationships were observed between averaged HRV measures and HRR. However, while interpreting the relationships found between HRV_AV4_ and HRR (30,1,2,3), the strength of these relationships were consistently weaker in averaged markers compared to HRV_DO_ (see Table 2). Specifically, for HRV_AV3_ the HRV_DO_ was not included in the average and demonstrated the fewest relationships. When considering HRV_AV4_, the relationships improved considerably, but are still less than that of HRV_DO_, leading the investigators to believe that this may be artifact driven by the strength of the HRV_DO_ measure and should be considered when interpreting the relationships found in the HRV averaged measures. This is in direct contrast to the hypothesis of this study as well as several prior studies utilizing single day measurements^12,13,29^. A potential explanation for this is pre-exercise anticipation, which has been shown to negatively alter HRV during resting periods^34^. When observing average HRV over the four measurements, the DO had the lowest values (Table 1), demonstrating this effect. Since this was a single bout of exercise, it cannot be determined if this had a deleterious effect on exercise performance.

When evaluating the various indices of HRV and HRR there are a wide array of populations and protocols utilized within the literature, and as such, there becomes a staggering number of variants that could be accounted for. This study was the first step towards a better understanding of the application of average HRV on ANS recovery, and was not an attempt to control for all of these variations found within the literature, and therefore came with inherent limitations. First, the participant population was a relatively homogenous sample of young, healthy females. Therefore, results cannot be applied to older adults, unhealthy populations, or males. Relationships between sex and race were not evaluated, and future studies should include both males and females. Additionally, future studies should include HRV measures collected over consecutive and nonconsecutive days, whereas our study is limited to the evaluation of HRV measures between nonconsecutive days.

## Conclusion

Resting heart rate measures displayed the most robust correlation to HRR values. Regarding HRV measures and HRR, HRV_DO_ demonstrated the strongest relationship to HRR and is likely the primary influencing factor of the observed correlations between HRR and HRV_AV4_. Therefore, the authors conclude that averaged 3–4 day HRV measures do not strengthen the prediction of cardiovascular recovery following a GXT within the confines of this study. Though mechanisms were not identified, these findings provide new and interesting insight into the relationship of HRR and resting HRV. With the continued application of HRV as tool for guided interventions, the understanding of the ANS and its relationship to recovery is paramount. The findings of this study are in line with those who found significant correlations between resting HRV values and HRR following a maximal exercise test, however the use of averaged (non-consecutive) measures of HRV may not provide further insight to aid in the explanation of equivocal findings throughout the literature^11–16,27,29^. Future research should include clinical populations due to differences in resting and stress related physiology. In addition, other physiological measures such as biomarkers of stress and metabolism to better reflect resting status, which will provide more insight into the system as a whole.

## Abbreviations

ANS: Autonomic nervous system
AV3: Heart rate variability averaged over the first three visits
AV4: Heart rate variability averaged over all four visits
DO: Heart rate variability measured the day of the graded exercise test (visit 4)
GXT: Graded Exercise Test
HRR: Heart Rate Recovery
HRR30: Heart Rate Recovery 30 s following graded exercise test
HRR1: Heart Rate Recovery 1 min following graded exercise test
HRR2: Heart Rate Recovery 2 min following graded exercise test
HRR3: Heart Rate Recovery 3 min following graded exercise test
HRR4: Heart Rate Recovery 4 min following graded exercise test
HRR5: Heart Rate Recovery 5 min following graded exercise test
HF: High frequency
HRV: Heart rate variability
LF: Low frequency
ln: Natural log
PNS: Parasympathetic nervous system
RMSSD: Root mean squared of successive R–R intervals
SDNN: Standard deviation of normal to normal intervals
SNS: Sympathetic nervous system
V1: First visit to the exercise physiology laboratory
V2: Second visit to the exercise physiology laboratory
V3: Third visit to the exercise physiology laboratory
V4: Fourth visit to the exercise physiology laboratory
VO_2peak_: Peak oxygen uptake
